# Investigation of Near-Infrared Spectroscopy for Assessing the Macroscopic Mechanical Properties of Cross-Linked Polyethylene During Thermal Aging

**DOI:** 10.3390/ma18030504

**Published:** 2025-01-22

**Authors:** Chenying Li, Xiao Tan, Liguo Liu, Wei Zhang, Qiming Yang, Jingying Cao, Enci Zhou, Mingzhen Li, Zaixin Song

**Affiliations:** 1 State Grid Jiangsu Electric Power Co., Ltd. Electric Power Research Institute, Nanjing 211103, China; chlichenying@163.com (C.L.); zw_xjtu@163.com (W.Z.); cjycjq@126.com (J.C.); 2 State Grid Jiangsu Electric Power Company, Nanjing 211103, China; 3 School of Electrical Engineering and Automation, Nantong University, Nantong 226019, China; 2412310013@stmail.ntu.edu.cn; 4 Department of Industrial and Systems Engineering, The Hong Kong Polytechnic University, Hong Kong, China

**Keywords:** XLPE cable, NIR, EAB, TS, correlation analysis, aging

## Abstract

The present study investigates the relationship between the near-infrared (NIR) spectral characteristics of cross-linked polyethylene (XLPE) insulation materials and their macroscopic properties, with the aim of establishing a reference framework for non-destructive material aging analysis. Accelerated thermal aging tests were conducted on samples of XLPE cables. These samples underwent Fourier-transform infrared spectroscopy (FTIR), elongation at break (EAB), and tensile strength (TS) tests. The temporal variation curves of the carbonyl index (CI), EAB, and TS were obtained at aging temperatures of 105 °C, 135 °C, 155 °C, and 180 °C. Additionally, NIR spectroscopy was performed on the aged XLPE samples, producing absorbance curves corresponding to different aging times at these temperatures. The absorption peaks of ‘C-H (-CH_2_-)’ (1730 nm/1764 nm) were analyzed to determine their temporal variation patterns. Finally, a correlation analysis was conducted between the NIR results and those of the FTIR, EAB, and TS tests, revealing numerical relationships between NIR characteristic peaks and FTIR, EAB, and TS data. These quantified correlations demonstrate that NIR can effectively represent macroscopic mechanical properties, thereby simplifying the procedures for monitoring material aging and providing valuable results without requiring destructive testing. Results indicate that there is a certain feasibility in replacing traditional cable aging tests with NIR.

## 1. Introduction

By December 2022, the State Grid Corporation of China (SGCC) had achieved a total of 990,000 km of transmission and distribution cables, reflecting a year-on-year increase of 14%. The urban-grid-cabling rate stood at 55.8%. Notably, the length of cables in service for over 15 years reached 110,000 km, highlighting the growing challenge of aging infrastructure [1,2,3]. From a global perspective, the global wire and cable market was valued at approximately $12.5 billion in 2021 and is projected to reach $19.2 billion by 2026, with a compound annual growth rate of around 9% [4]. In future power systems, cross-linked polyethylene (XLPE) cables are expected to remain extensively utilized in transmission and distribution networks, owing to their superior electrical properties and dependable operational stability. Over extended periods of operation, cables can be influenced by factors, such as temperature variations, mechanical stress, electrical aging, and external environmental conditions. These influences significantly affect the macroscopic mechanical properties of the cables, including their strength, toughness, and flexibility, which in turn play a crucial role in determining their operational durability and safety. Therefore, employing efficient and non-destructive methods to assess the mechanical properties of XLPE cables is of significant importance for the safe, stable, and economical operation of future power systems.

Multiple studies have shown that the insulation aging of XLPE cables is not the result of a single stress factor but rather the combined effect of multiple factors, such as electrical, thermal, and environmental influences [5,6,7]. The aging and deterioration of cable insulation in operation has always been a focus of attention for power companies, but currently, there is still relatively little research on the deterioration performance of the insulation materials of in-service XLPE cables. Therefore, it is essential to measure the changes in the electrical and physicochemical properties of XLPE cable insulation before and after aging, and to study the corresponding relationships between the various properties of the cable insulation materials and their aging, in order to assess their aging status.

The aging process of XLPE cables is challenging to observe directly and necessitates the use of various analytical techniques for investigation. Due to the simultaneous influence of electric fields, temperature variations, mechanical stresses, and environmental conditions, the dielectric and physicochemical properties, as well as the microscopic structure of the insulation, experience alterations. These modifications can, in turn, have a direct or indirect impact on the electrical and mechanical properties of the material. A comparison of the dielectric and physicochemical properties of insulation materials with varying operational and aging durations facilitates the evaluation of their aging condition [8,9,10]. Meanwhile, the signs of aging are diverse, encompassing alterations in properties such as the melting point, infrared carbonyl absorbance, oxidation induction time, electrical strength, tensile strength, and elongation at break (EAB). In [11], a comprehensive analysis was conducted using various techniques, including Fourier Transform Infrared Spectroscopy (FTIR), Electron Spin Resonance (ESR), Thermogravimetric Analysis (TGA), Differential Scanning Calorimetry (DSC), contact angle measurements, and Scanning Electron Microscopy (SEM). These methods were applied to both artificially aged and non-aged XLPE samples. The findings suggest that these tests are effective tools for evaluating the aging condition of the XLPE. However, the paper did not analyze the correlations between the various test results, which leads to certain limitations in the generalization of the findings. In [12], FTIR, X-ray Diffraction (XRD), and EAB tests were employed to analyze the chemical characteristics of XLPE cables. The study also examined the interrelationships between the physical, chemical, and mechanical properties of the insulation materials. While the study primarily simulated the actual operating environment through thermal oxidative aging tests, it did not fully consider other influencing factors, such as mechanical stress and humidity variations, which could affect the comprehensiveness of the results. In [13], FTIR, XRD, and Ultraviolet–Visible (UV–Vis) spectrophotometry were employed to study the thermal degradation of XLPE insulation, with a particular focus on the effects of thermal aging. However, the paper also did not analyze the correlations between the various test results, making it impossible to generalize the findings across different test results. In [14], FTIR, DSC, EAB, and indenter modulus (IM) spectra were used for assessing the aging condition. The paper discussed the correlation between EAB and the integrated absorption of carbonyls, but the correlations of other indicators were not analyzed in depth.

The aforementioned research suggests that the progression of aging can be observed through variations in several testing parameters. These parameters include macroscopic properties such as tensile strength (TS) and EAB, as well as properties linked to the material’s physicochemical characteristics, such as those identified through FTIR analysis. While the tests referenced typically necessitate destructive testing to provide a thorough evaluation of aging, such testing conditions are often not feasible in most engineering applications. The primary objective of this paper is to investigate the relationships between near-infrared spectroscopy (NIR) and the macroscopic material properties in greater detail, with the goal of identifying and quantifying these correlations as accurately as possible. By establishing these quantitative relationships, this study seeks to streamline the process of aging diagnosis, enabling the extraction of valuable insights without the necessity for destructive testing. This approach not only enhances efficiency but also offers a non-invasive alternative for monitoring material aging in practical applications. Non-destructive, in situ NIR testing will represent a completely new application scenario, where aging tests of cables can maintain the integrity of samples, extend their lifespan, be efficient and cost-effective, and provide continuous monitoring capabilities. In addition, NIR measurements are ideally for in situ measurements, which provide real-time data from a material’s actual operating environment, enabling more accurate assessment of its performance and condition under real-world conditions.

## 2. Materials and Methods

**Figure 1:**
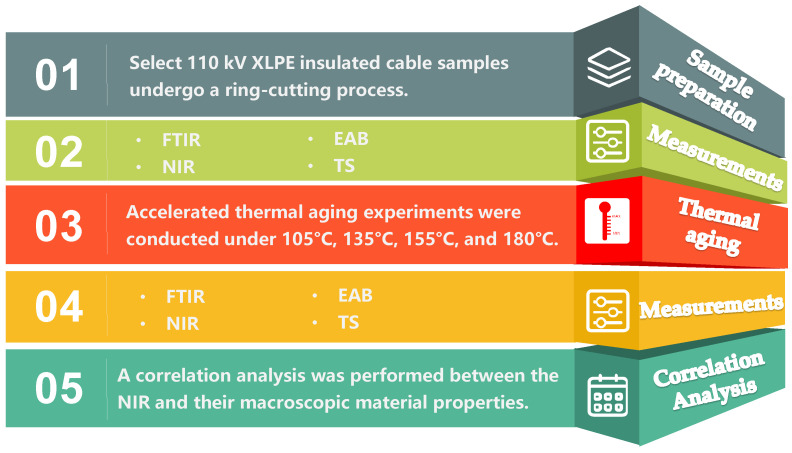
Flowchart of the research analysis procedure.

A flowchart illustrating the methodology of this study is presented in Figure 1. The process begins with the preparation of samples, specifically utilizing 110 kV XLPE cables to create ring-cut specimens. Following sample preparation, a series of tests—namely FTIR, EAB, TS, and NIR—are conducted to obtain initial results, which are meticulously recorded. Subsequently, the prepared samples undergo accelerated thermal aging tests at specified temperatures of 105 °C, 135 °C, 155 °C, and 180 °C. The accelerated thermal aging tests were conducted, following the guidelines outlined in IEC 60811-401 [15], ensuring consistency and reproducibility of results. This step is crucial for simulating long-term aging effects and leads to the acquisition of aged samples. After the aging process, a second round of FTIR, EAB, TS, and NIR testing is performed on these aged samples, with results again being systematically documented. Finally, a comprehensive correlation analysis is carried out to explore the relationships between the NIR data and the results obtained from the other testing methods. This analysis aims to establish numerical correlations that may enhance our understanding of the mechanical properties of the aged XLPE cables and their potential implications for future applications in power systems.

(1) Sample preparation

**Figure 2:**
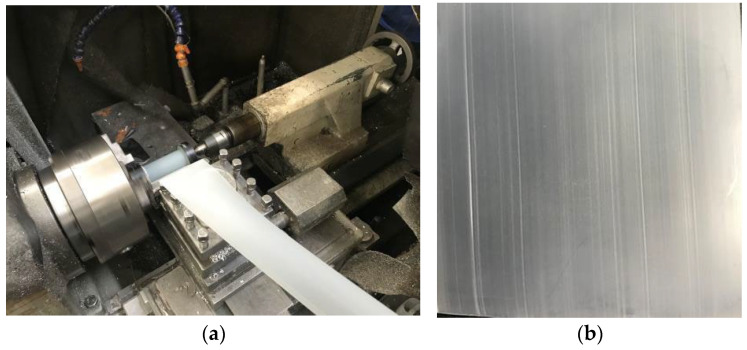
Ring-cutting process of XLPE cable: (**a**) the ring-cutting process; (**b**) XLPE samples.

Initially, we selected 110 kV XLPE-insulated cable samples that had an operational age of 0 years. These samples underwent a ring-cutting process, yielding XLPE slices measuring 10 mm × 10 mm and approximately 0.5 mm in thickness, with uniformity achieved through precision machine cutting, as shown in Figure 2. Then, the ring-cut XLPE insulation samples were used for subsequent thermal oxidative aging tests. After preparation, the slices were placed in a controlled oven for aging. The oven temperature was maintained within a tolerance of ±1 °C of the set point, and the exhaust rate was kept constant throughout the aging process to ensure consistency and reliability of the results.

(2) FTIR testing

Subsequently, the FTIR spectroscopy was conducted on the cable insulation samples using a Nicolet iN10 spectrometer (Thermo Fisher Scientific, Waltham, MA, USA). The analysis was performed in attenuated total reflectance (ATR) mode, covering a wavenumber range from 4000 to 500 cm^−1^, with a resolution of 4 cm^−1^. During the aging process, the combined effects of heat and oxygen cause XLPE to generate by-products containing oxygenated functional groups, such as carbonyls. These by-products are primarily concentrated in the wavenumber range between 1630 cm^−1^ and 1770 cm^−1^. To quantitatively assess the variation in carbonyl content within the XLPE samples, a carbonyl index was employed. This index serves as a measure of the changes in the thermal oxidative aging products of XLPE over time, providing a quantitative description of the aging process. The limitation of FTIR testing mainly lies in the fact that sample preparation requires slicing, which is a destructive test. For cables, the results lack reproducibility.

(3) EAB and TS testing

The mechanical tests were conducted following the guidelines outlined in IEC 60811-501 [16]. EAB and TS are fundamental mechanical properties that serve as key indicators of a material’s overall mechanical performance. These parameters provide a macroscopic assessment of characteristics such as strength and plasticity, while also offering insights into the material’s microscopic structure, including its crystalline arrangement and potential internal defects. To evaluate these properties, tensile tests were performed on cable insulation samples subjected to varying degrees of aging. The tests were conducted using a 5KNCMT-4503 electronic tensile testing machine, manufactured by Meester Industrial Co., Ltd. (Chico, CA, USA), with dumbbell-shaped samples and a constant tensile rate of 100 mm/min.

The main limitations of the EAB and TS tests are as follows: (1) deviations in shape, size, and edge defects during sample preparation may affect the accuracy of the results; (2) sliding of the fixtures and alignment errors may result in improper fixation of the sample or misalignment, leading to uneven stress distribution; (3) a testing rate that is too fast or too slow may cause the test results to deviate from true performance. To mitigate these limiting factors, the paper employs the following methods: (1) standardized sample preparation to ensure consistency in shape, size, and edges; (2) proper clamping and alignment using non-slip fixtures and an automatic centering device to reduce errors; (3) selecting an appropriate testing rate.

(4) NIR testing

NIR testing of the XLPE samples was analyzed using the UV-3600 spectrophotometer (UV-VIR-NIR Spectrophotometer) manufactured by Shimadzu Corporation in Kyoto, Japan. The instrument was configured to operate in transmission mode, covering a wavelength range from 1600 to 1900 nm. The testing procedure involved a sampling interval of 1 nm and a resolution of 0.17 nm to ensure precise measurement. For reference, the baseline signal was established by recording the response obtained in the absence of any sample. This method allowed for accurate characterization of the spectral properties of the insulation materials.

The main limitation of NIR is that the penetration depth of NIR light is limited, making it difficult to detect the internal components of thick samples. Since the paper uses the same samples as previous tests, there is no issue with penetration depth. During on-site testing, diffuse reflection techniques can be considered for use to increase the detection depth.

(5) Accelerated thermal aging

Accelerated thermal aging experiments were conducted, selecting four aging temperatures: 105 °C, 135 °C, 155 °C, and 180 °C. Multiple parameter measurements were performed on thermal aging samples at different temperatures and times to extract aging-related parameters, thereby assessing the aging status of the cable insulation. The specific temperature and time schedule for the experiments is shown in Table 1.

(6) Correlation analysis

Finally, a correlation analysis was conducted to examine the relationship between the NIR results of thermally oxidized XLPE samples and their macroscopic material properties. This involved fitting an equation to describe the changes in key parameters over aging time and performing a detailed analysis of the associated indicators. The findings from the FTIR, EAB, and TS tests, along with the results of the correlation analysis, will be discussed in the following section.

## 3. Results

### 3.1. Results of FTIR Testing and Mechanical Performance Testing

#### 3.1.1. Variation Pattern of the Carbonyl Index in XLPE Cable Insulation

**Figure 3:**
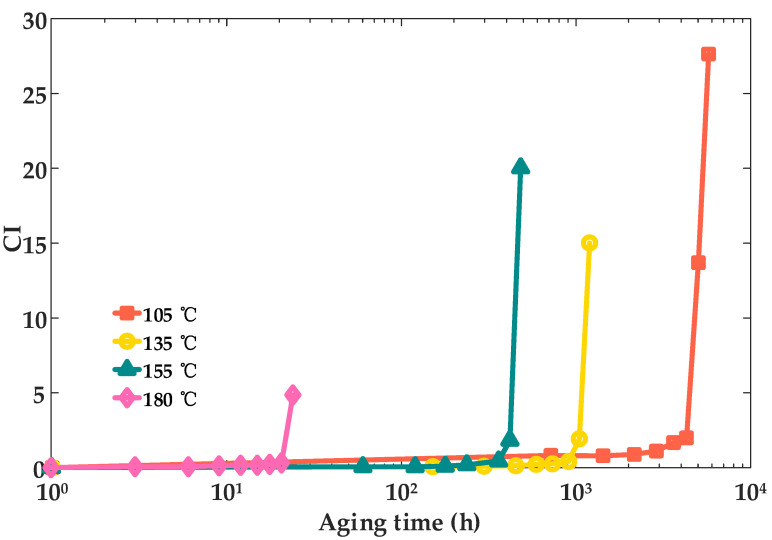
Variation curves of the carbonyl index of XLPE cable insulation with thermal aging time.

In order to study the variation pattern of the carbonyl index (CI) in XLPE cable insulation, the FTIR test was carried out. CI is determined based on the ratio of the intensity of the carbonyl characteristic absorption peak to that of the reference group in the infrared spectrum of the sample. This process is automatically calculated by the software during the instrument testing. Figure 3 illustrates the relationship between the carbonyl index and aging time for cable insulation samples subjected to thermal aging at temperatures of 105 °C, 135 °C, 155 °C, and 180 °C. The curves provide a comprehensive depiction of how the carbonyl index evolves over time under varying thermal conditions, reflecting the progression of oxidative aging in the material.

Based on the data presented in Figure 3, the carbonyl index (CI) of the samples exhibits a gradual increase as aging progresses. Moreover, under the four test temperatures, the CI curve exhibited a significant sudden increase at the end. This observation indicates that thermal aging leads to a reduction in the absorption peak associated with -CH_2_ groups, implying degradation and disruption of the original molecular chains in the material [17]. Furthermore, the carbonyl index demonstrates a distinct trend: it increases slowly during the initial stages of aging, followed by a pronounced acceleration as the aging time extends.

#### 3.1.2. Variation Pattern of Elongation at Break with Aging Time

**Figure 4:**
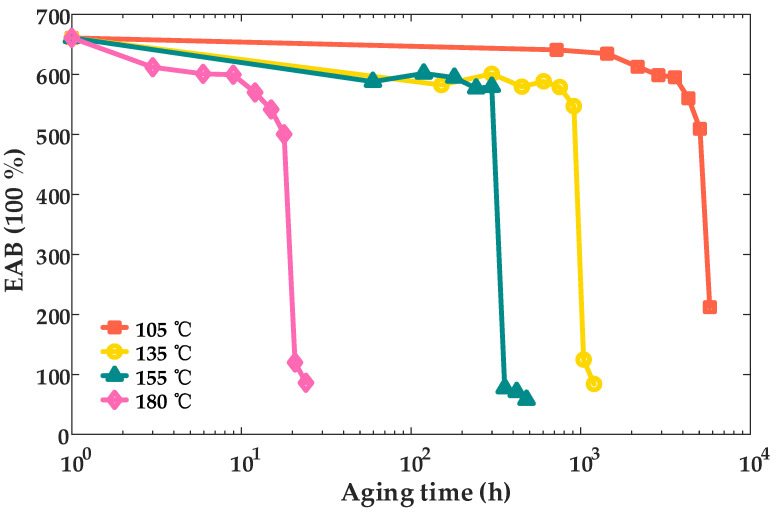
Variation curves of elongation at break of XLPE cable insulation with aging time.

In order to study the variation pattern of the EAB with aging time, the EAB test was carried out. Figure 4 presents the EAB test results for XLPE cable insulation samples subjected to thermal aging at temperatures of 105 °C, 135 °C, 155 °C, and 180 °C. These results provide valuable insights into the mechanical performance of the material under varying thermal aging conditions, highlighting the impact of elevated temperatures on the material’s elasticity and durability.

The test results reveal that the elongation at break (EAB) exhibits a consistent variation pattern across all four groups of XLPE cable insulation samples aged at temperatures of 105 °C, 135 °C, 155 °C, and 180 °C. During the initial stages of aging, the EAB decreases gradually, indicating a relatively slow decline in the material’s elasticity. However, after a certain aging threshold, the EAB undergoes a rapid and pronounced reduction, signifying a critical stage in the degradation process where the material’s mechanical properties deteriorate sharply.

#### 3.1.3. Variation Pattern of Tensile Strength with Aging Time

**Figure 5:**
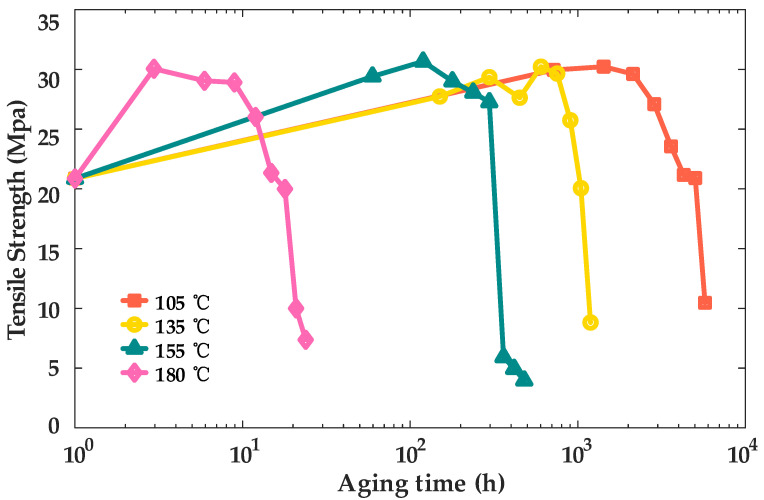
Variation curves of tensile strength of XLPE cable insulation with aging time.

In order to study the variation pattern of the TS with aging time, the TS test was carried out. Figure 5 presents the tensile strength test results for XLPE cable insulation samples subjected to thermal aging at temperatures of 105 °C, 135 °C, 155 °C, and 180 °C. These results provide critical insights into the mechanical strength and structural integrity of the material under prolonged exposure to elevated temperatures, illustrating the effects of thermal aging on its tensile performance.

The tensile test results demonstrate a distinct trend in the tensile strength of the material, characterized by an initial gradual increase followed by a sharp decline. The initial increase in tensile strength can be attributed to recrystallization processes, which lead to an increased number of chain segments within the crystalline regions of the material [18]. However, as aging progresses, the tensile strength decreases significantly, primarily due to the degradation and breaking of molecular chains, which compromise the structural integrity of the material.

### 3.2. Results of the Near-Infrared Spectroscopy Testing

#### 3.2.1. Evolution Pattern of the Near-Infrared Spectroscopy of XLPE Samples with Aging Time

The near-infrared spectroscopy of the insulation material samples was analyzed using a UV-3600 UV-VIS-NIR spectrophotometer, manufactured by Shimadzu Corporation, Japan. The measurements were conducted in transmission mode, covering a wavelength range from 1600 to 1900 nm, with a sampling interval of 1 nm and a spectral resolution of 0.17 nm. The reference signal was obtained by recording the instrument’s response without any sample in place, ensuring accurate baseline calibration for the spectral analysis.

**Figure 6:**
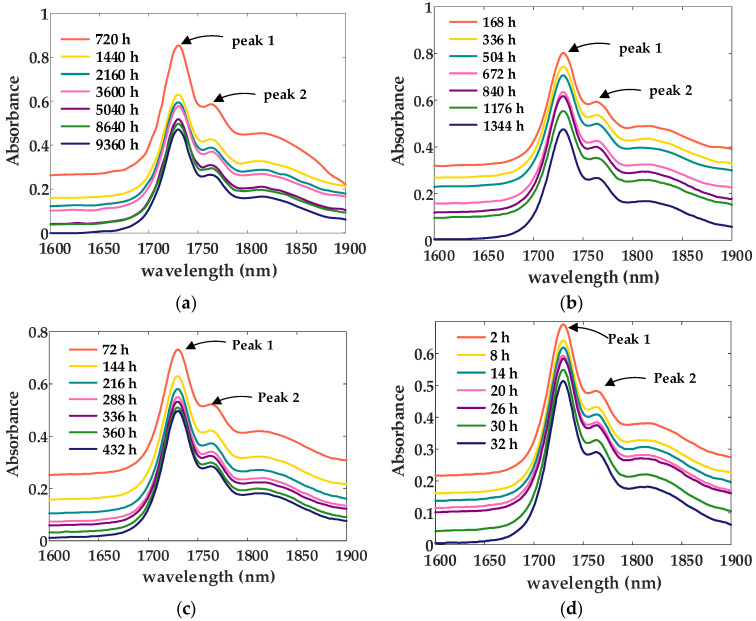
Variation curves of the near-infrared spectroscopy of XLPE insulation at the same aging temperature: (**a**) 105 °C; (**b**) 135 °C; (**c**) 155 °C; (**d**) 180 °C.

As presented in Figure 6, at a given aging temperature, the absorbance of the characteristic peaks in the NIR of XLPE decreases with increasing aging time. This phenomenon can be attributed to the breaking of molecular chains in the XLPE samples due to thermal oxidative aging [19]. The two characteristic peaks observed at 1730 nm and 1764 nm correspond to the first harmonic frequencies of the asymmetric and symmetric vibrations of methylene (C-H) bonds, respectively. With the progression of aging, the absorbance in both spectral regions decrease. Additionally, the spectral bands at 1710 nm and 1762 nm correspond to short helical molecular segments in the amorphous region of XLPE, while the bands at 1730 nm and 1765 nm correspond to long helical molecular segments in the crystalline region of XLPE [20]. From the trends presented in Figure 6, it can be observed that as the aging extent increases, the absorbance of the characteristic peaks corresponding to the crystalline region gradually decreases, whereas the absorbance of the characteristic peaks in the amorphous region gradually increases. This phenomenon indicates that as aging progresses, the molecular chains in the crystalline region break during thermal oxidative aging and transition into the amorphous region, resulting in a transformation from the crystalline region to the amorphous region [21].

#### 3.2.2. Characteristic Analysis of the Near-Infrared Spectroscopy Curves

At elevated temperatures, XLPE undergoes thermal oxidative degradation, resulting in alterations to its aggregated structure and physical properties. The thermal oxidative degradation process is primarily driven by autoxidation, which can be divided into three distinct stages: initiation, propagation, and termination. During the initiation phase, the segments of XLPE macromolecular chains with relatively lower bond energies are the first to break, leading to the formation of free radicals [22]. These free radicals serve as the primary reactants that trigger subsequent degradation processes. In the propagation stage, the free radicals formed during initiation undergo oxidation reactions, resulting in the formation of peroxide radicals. These peroxide radicals, in turn, interact with the XLPE macromolecular chains, leading to the production of peroxides [23]. This chain reaction sustains the degradation process, progressively altering the molecular structure and accelerating the thermal oxidative breakdown of the material. At elevated temperatures, the peroxides undergo decomposition, giving rise to new free radicals. These newly generated free radicals can further interact with the XLPE macromolecular chains, initiating additional reactions that result in the formation of more free radicals and peroxides [24]. This continuous cycle of decomposition and regeneration perpetuates the thermal oxidative degradation process, progressively compromising the structural integrity of the material. Each reaction generates free radicals and peroxides that actively participate in oxidation reactions, leading to a self-sustaining and progressively accelerated oxidation process. In the termination phase, free radicals and peroxide radicals collide and undergo bimolecular coupling reactions, resulting in the formation of new crosslinking bonds, which effectively halt the thermal oxidative aging process. Throughout this aging process, thermal oxidative reactions cause the degradation of XLPE macromolecular chains, leading to chain scission and the formation of smaller molecular fragments and carbonyl compounds as byproducts. These degradation products disrupt the aggregated structure of XLPE, inducing changes in its crystalline and amorphous phases, which ultimately deteriorates the material’s mechanical properties and compromises the performance of the insulation. The absorption peaks corresponding to the ‘C-H (-CH_2_-)’ bonds, located at 1730 nm and 1764 nm in the NIR of XLPE, provide insights into the relative quantity and integrity of the molecular chain segments, serving as indicators of the material’s aging state. As thermal oxidative degradation progresses, the breakdown of XLPE macromolecular chains leads to a reduction in the concentration and structural continuity of these chain segments. Consequently, the absorbance intensity of the characteristic peaks in the NIR spectrum diminishes progressively with increasing aging time, reflecting the material’s degradation and loss of structural integrity.

**Figure 7:**
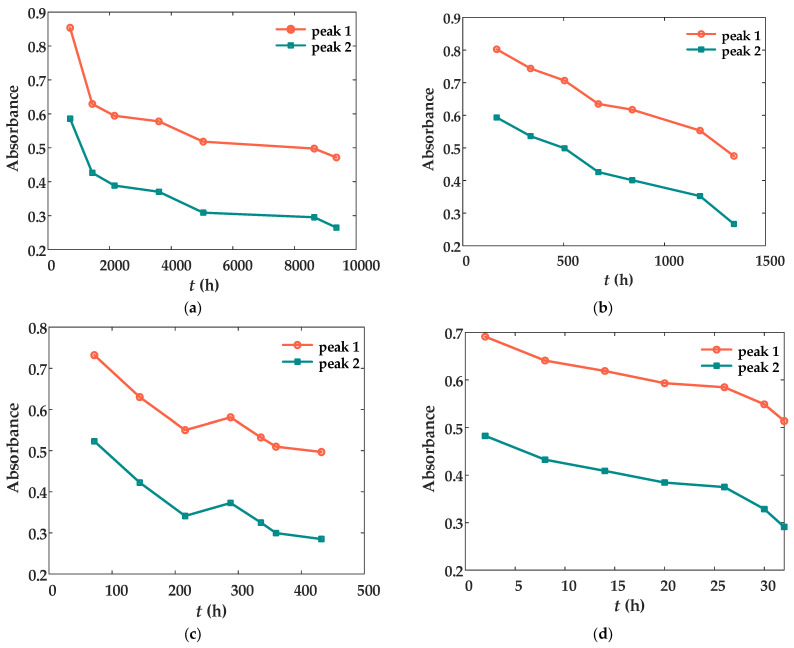
Variation curves of the near-infrared spectroscopy characteristic peak values with time at the same aging temperature: (**a**) 105 °C; (**b**) 135 °C; (**c**) 155 °C; (**d**) 180 °C.

The absorption peaks corresponding to the ‘C-H (-CH_2_-)’ bonds at 1730 nm and 1764 nm in the NIR of XLPE represent the two most prominent peaks, as illustrated in Figure 6. Given their significant amplitude, these peaks can be selected as characteristic indicators for further analysis. The relationship between the amplitudes of these characteristic peaks and aging time has been systematically examined, with the resulting findings presented in Figure 7. This analysis provides a quantitative basis for evaluating the aging progression of XLPE materials.

As illustrated in Figure 7, at all four test temperatures, the peak values of the two selected characteristic absorption peaks generally exhibit a decreasing trend with increasing aging time. However, a slight deviation from monotonicity is observed in Figure 7c, which can be attributed to factors such as short aging-time intervals and minor variations in the material’s internal consistency. Furthermore, the functional equations describing the relationship between aging time and the peak values for each group of curves can be derived from Figure 7, as presented in Equations (1)–(4). These equations were obtained using curve-fitting techniques [25,26,27], where an exponential fitting model was applied. The least squares method was utilized to minimize the sum of squared errors between the experimental data and the predicted values, ensuring a robust representation of the observed trends.


(1) $$\begin{array}{c} y_{105p1}=2.0604\times e^{\left(-0.003t\right)}+0.6286\\ y_{105p2}=0.9556\times e^{\left(-0.0023t\right)}+0.4220 \end{array}$$

(2) $$\begin{array}{c} y_{135p1}=0.8582\times e^{\left(-0.0004t\right)}\\ y_{135p2}=0.6598\times e^{\left(-0.0006t\right)} \end{array}$$

(3) $$\begin{array}{c} y_{155p1}=0.4793\times e^{\left(-0.0211t\right)}+0.6567\times e^{\left(-0.0006t\right)}\\ y_{155p2}=0.0012\times e^{\left(-2.8837t\right)}+0.3472\times e^{\left(-0.1399t\right)} \end{array}$$

(4) $$\begin{array}{c} y_{180p1}=0.7369\times e^{\left(-{\left(\frac{t+11.7953}{52.5193}\right)}^{2}\right)}+0.1555\times e^{\left(-{\left(\frac{t-29.3104}{12.2498}\right)}^{2}\right)}\\ y_{180p2}=0.5142\times e^{\left(-{\left(\frac{t+8.0465}{38.4590}\right)}^{2}\right)}+0.1404\times e^{\left(-{\left(\frac{t-27.5459}{11.0308}\right)}^{2}\right)} \end{array}$$

In Equations (1)–(4), *t* represents the aging time, *y*_105*p*1_ represents the absorbance value of peak 1 at aging temperature 105 °C, *y*_105*p*2_ represents the absorbance value of peak 2 at aging temperature 105 °C, *y*_135*p*1_ represents the absorbance value of peak 1 at aging temperature 135 °C, *y*_135*p*2_ represents the absorbance value of peak 2 at aging temperature 135 °C, *y*_155*p*1_ represents the absorbance value of peak 1 at aging temperature 155 °C, *y*_155*p*2_ represents the absorbance value of peak 2 at aging temperature 155 °C, *y*_180*p*1_ represents the absorbance value of peak 1 at aging temperature 180 °C, and *y*_180*p*2_ represents the absorbance value of peak 2 at aging temperature 180 °C. It should be noted that Equations (1)–(4) represent fitted mathematical expressions and do not inherently carry explicit physical or chemical significance. Nevertheless, the coefficients of determination (R^2^) for each fitted equation exceed 0.95, indicating a high degree of accuracy in describing the data trends. The primary purpose of these equations is to provide a simplified and quantitative representation of the four sets of curves depicted in Figure 5, enabling the observed relationships to be reproduced in a clear and concise mathematical form.

### 3.3. Expressions of the Macroscopic Mechanical Properties of XLPE

In order to facilitate a more intuitive investigation of the relationship between the NIR and the macroscopic mechanical properties of XLPE, the curves for the CI, EAB, and TS, as discussed in Section 3.1, were subjected to curve fitting. The corresponding results are presented in Equations (5)–(7). Various mathematical models, including exponential, Gaussian, and polynomial models, were employed to achieve the curve fitting, ensuring an accurate representation of the observed trends across the data sets.

(5) $$\begin{array}{c} CI_{105}=21.5742\times e^{\left(9.6420\times 10^{-4}t\right)}-21.6087\times e^{\left(9.6303\times 10^{-4}t\right)}\\ CI_{135}=0.0578\times e^{\left(0.0017t\right)}+3.1028\times 10^{-7}\times e^{\left(0.0148t\right)}\\ CI_{155}=0.0343\times e^{\left(0.0063t\right)}+3.5757\times 10^{-8}\times e^{\left(0.0417t\right)}\\ CI_{180}=8.624\times 10^{-16}\cdot e^{\left(1.5133t\right)}+0.0426\times e^{\left(0.0932t\right)} \end{array}$$

(6) $$\begin{array}{c} EAB_{105}=-0.0001\times e^{\left(0.0025t\right)}+659.4\\ EAB_{135}=-1.4326\times 10^{-6}\cdot t^{3}-0.0020t^{2}-0.7756t+661.5020\\ EAB_{155}=-1.3916\times 10^{-5}\cdot t^{3}+0.0062t^{2}-1.0426t+661.7644\\ EAB_{180}=-0.1228t^{3}+3.0897t^{2}-28.3824t+686.1439 \end{array}$$

(7) $$\begin{array}{c} TS_{105}=31.0578\times e^{\left(-{\left(\frac{t-1761}{3047}\right)}^{2}\right)}+10.8555\times e^{\left(-{\left(\frac{t-4961}{876.6}\right)}^{2}\right)}\\ TS_{135}=28.1209\times e^{\left(-{\left(\frac{t-790.2}{391.4}\right)}^{2}\right)}+25.9016\times e^{\left(-{\left(\frac{t-176.8}{367.7}\right)}^{2}\right)}\\ TS_{155}=1.102\times 10^{-6}\cdot t^{3}-0.001056t^{2}+0.2161t+19.93\\ TS_{180}=29.6834\times e^{\left(-{\left(\frac{t-7.5912}{13.6927}\right)}^{2}\right)} \end{array}$$

In Equations (5)–(7), *t* represents the aging time, *CI*_105_ represents the carbonyl index at aging temperature 105 °C, *CI*_135_ represents the carbonyl index at aging temperature 135 °C, *CI*_155_ represents the carbonyl index at aging temperature 155 °C, *CI*_180_ represents the carbonyl index at aging temperature 180 °C, *EAB*_105_ represents the elongation at break at aging temperature 105 °C, *EAB*_135_ represents the elongation at break at aging temperature 135 °C, *EAB*_155_ represents the elongation at break at aging temperature 155 °C, *EAB*_180_ represents the elongation at break at aging temperature 180 °C, *TS*_105_ represents the tensile strength at aging temperature 105 °C, *TS*_135_ represents the tensile strength at aging temperature 135 °C, *TS*_155_ represents the tensile strength at aging temperature 155 °C, and *TS*_180_ represents the tensile strength at aging temperature 180 °C. Again, Equations (5)–(7) are merely fitted expressions and do not necessarily have explicit physical or chemical meanings, the coefficient of determination (R^2^) of each fitting are above 0.9.

### 3.4. Correlation Between the Near-Infrared Spectroscopy and Macroscopic Material Properties

In conclusion, Equations (1)–(7) all share the same variable *t*, which represents the aging time in the thermal aging tests. It is important to note that, while *t* may correspond to different temperatures within the experiments, these thermal aging conditions do not directly reflect real-world natural aging, making direct temporal comparisons between them difficult. Nevertheless, the variable *t* serves as a crucial link, connecting the characteristic peak values of the near-infrared spectroscopy with the macroscopic material properties. This enables the formulation of a system of equations, even though they are based solely on the conditions of thermal aging tests. Thus, the correlation expressions can be derived by solving the system of Equations (1)–(7), which were shown in Equations (8)–(10), providing a mathematical framework to describe the relationship between aging time, spectral characteristics, and material properties.

(8) $$\begin{array}{c} CI_{105}=1.1236\times 10^{19}\times e^{\left(-76.5443\cdot y_{105p1}\right)}\\ CI_{105}=-0.0226\times e^{\left(0.3624\cdot y_{105p2}\right)}-2.5131\times 10^{12}\times e^{\left(-78.4318\cdot y_{105p2}\right)}\\ CI_{135}=6.5404\times 10^{15}\times e^{\left(-63.8239\cdot y_{135p1}\right)}-64.0272\times e^{\left(-8.1052\cdot y_{135p1}\right)}\\ CI_{135}=6.3412\times 10^{12}\times e^{\left(-84.1877\cdot y_{135p2}\right)}+2.1023\times 10^{4}\times e^{\left(-28.2098\cdot y_{135p2}\right)}\\ CI_{155}=4.1263\times 10^{17}\times e^{\left(-82.6537\cdot y_{155p1}\right)}\\ CI_{155}=1.9250\times 10^{12}\times e^{\left(-101.4290\cdot y_{155p2}\right)}+63.1479\times e^{\left(-16.2356\cdot y_{155p2}\right)}\\ CI_{180}=2.2288\times 10^{27}\cdot e^{\left(-95.4543\cdot y_{180p1}\right)}\\ CI_{180}=1.1314\times 10^{17}\cdot e^{\left(-87.0826\cdot y_{180p2}\right)} \end{array}$$

(9) $$\begin{array}{c} EAB_{105}=654.4973\times e^{\left(-0.0038\cdot y_{105p1}\right)}-8.7465\times 10^{13}\times e^{\left(-49.4322\cdot y_{105p1}\right)}\\ EAB_{105}=611.6848\times e^{\left(0.0668\cdot y_{105p2}\right)}-8.6238\times 10^{13}\times e^{\left(-81.2691\cdot y_{105p2}\right)}\\ EAB_{135}=733.5565\times e^{\left(-0.2618\cdot y_{135p1}\right)}-2.2974\times 10^{7}\times e^{\left(-20.2980\cdot y_{135p1}\right)}\\ EAB_{135}=656.2546\times e^{\left(-0.1672\cdot y_{135p2}\right)}-8.5406\times 10^{5}\times e^{\left(-23.1414\cdot y_{135p2}\right)}\\ EAB_{155}=644.8670\times e^{\left(0.1999\cdot y_{155p1}\right)}-4.4644\times 10^{6}\times e^{\left(-19.7995\cdot y_{155p1}\right)}\\ EAB_{155}=545.2824\times e^{\left(0.2719\cdot y_{155p2}\right)}-1.5821\times 10^{5}\times e^{\left(-22.7320\cdot y_{155p2}\right)}\\ \begin{array}{ll} EAB_{180} & =-4.9326\times 10^{9}\cdot {y_{180p1}}^{6}+1.8055\times 10^{10}\cdot {y_{180p1}}^{5}-2.7462\times 10^{10}\cdot {y_{180p1}}^{4}+2.2216\times 10^{10}\cdot {y_{180p1}}^{3}\\ & -1.0082\times 10^{10}\cdot {y_{180p1}}^{2}+2.4332\times 10^{9}\cdot y_{180p1}-2.4400\times 10^{8} \end{array}\\ \begin{array}{ll} EAB_{180} & =-2.2428\times 10^{9}\cdot {y_{180p2}}^{6}+5.4071\times 10^{9}\cdot {y_{180p2}}^{5}-5.3885\times 10^{9}\cdot {y_{180p2}}^{4}+2.8403\times 10^{9}\cdot {y_{180p2}}^{3}\\ & -8.3500\times 10^{8}\cdot {y_{180p2}}^{2}+1.2980\times 10^{8}\cdot y_{180p2}-8.3373\times 10^{6} \end{array} \end{array}$$

(10) $$\begin{array}{c} TS_{105}=-3.1267\times 10^{9}\times e^{\left(-35.6857\cdot y_{105p1}\right)}+34.7953\times e^{\left(-0.2145\cdot y_{105p1}\right)}\\ TS_{105}=-7.6131\times 10^{5}\times e^{\left(-32.7464\cdot y_{105p2}\right)}+37.1126\times e^{\left(-0.4406\cdot y_{105p2}\right)}\\ TS_{135}=20.9266\times e^{\left(-{\left(\frac{y_{135p1}-0.6076}{0.0835}\right)}^{2}\right)}+28.8115\times e^{\left(-{\left(\frac{y_{135p1}-0.7755}{0.1469}\right)}^{2}\right)}\\ TS_{135}=19.0265\times e^{\left(-{\left(\frac{y_{135p2}-0.3978}{0.0768}\right)}^{2}\right)}+29.2241\times e^{\left(-{\left(\frac{y_{135p2}-0.5650}{0.1612}\right)}^{2}\right)}\\ TS_{155}=1.0083\times 10^{4}\cdot {y_{155p1}}^{4}-2.7562\times 10^{4}\cdot {y_{155p1}}^{3}+2.6854\times 10^{4}\cdot {y_{155p1}}^{2}-1.1029\times 10^{4}\cdot y_{155p1}+1.6249\times 10^{3}\\ TS_{155}=1.2298\times 10^{4}\cdot {y_{155p1}}^{4}-2.1879\times 10^{4}\cdot {y_{155p2}}^{3}+1.3385\times 10^{4}\cdot {y_{155p2}}^{2}-3.2374\times 10^{3}\cdot y_{155p2}+268.6370\\ TS_{180}=-1.2764\times 10^{6}\cdot {y_{180p1}}^{4}+3.3486\times 10^{6}\cdot {y_{180p1}}^{3}-3.2954\times 10^{6}\cdot {y_{180p1}}^{2}+1.4417\times 10^{6}\cdot y_{180p1}-2.3657\times 10^{5}\\ TS_{180}=-1.5551\times 10^{6}\cdot {y_{180p2}}^{4}+2.7782\times 10^{6}\cdot {y_{180p2}}^{3}-1.8615\times 10^{6}\cdot {y_{180p2}}^{2}+5.5441\times 10^{5}\cdot y_{180p2}-6.1894\times 10^{4} \end{array}$$

The symbols in Equations (8)–(10) have the same meanings as those in Equations (1)–(7). With the bridging of aging time *t*, the carbonyl index *CI*, elongation at break *EAB*, and tensile strength *TS* can all be represented by the two characteristic peak values of the NIR. Given that Equations (1)–(7) are purely mathematical expressions without direct physical interpretation, Equations (8)–(10) similarly serve as representations of the mathematical relationships. The apparent exaggeration of some coefficients in Equations (8)–(10) can be attributed to the inflection points present in the relationship curves, which exhibit significant curvature. These inflection points cause variations in the rate of change, leading to larger coefficients in the fitting equations to accurately capture the complex behavior of the curves.

## 4. Discussion

From the result curves presented in Section 3, it is evident that as the aging time progresses, all tests demonstrate varying degrees of change in their outcomes, with consistent patterns observed across the different tests. Notably, in the tests for the *CI*, *EAB*, and *TS,* the indicators show a marked shift after XLPE reaches a certain stage of aging. In contrast, the changes in the characteristic curves and peak values from the NIR tests occur at a more gradual rate. As a result, the relationships between the amplitudes of the NIR peaks and the macroscopic properties of *CI*, *EAB*, and *TS* more prominently capture the sharp transitions observed in the mechanical property tests, highlighting the more abrupt nature of the changes in these tests.

Furthermore, cosine similarity [28] can be used to analyze the similarities among the FTIR, EAB, and TS test results (waveforms in Figure 3, Figure 4 and Figure 5). It measures the similarity between two vectors by calculating the cosine of the angle between them, and its expression can be presented in Equation (11):

(11) $$\cos\left(\theta \right)=\frac{x\cdot y}{\left\|x\right\|\left\|y\right\|}=\frac{\sum_{i=1}^{n}\left(x_{i},y_{i}\right)}{\sqrt{\sum_{i=1}^{n}x_{i}^{2}}\sqrt{\sum_{i=1}^{n}y_{i}^{2}}}$$

Among them, **x** = [*x*_1_, *x*_2_, …, *x_n_*], **y** = [*y*_1_, *y*_2_, …, *y_n_*], **x** and **y** are two vectors, and *n* is the number of sampling points of the vectors. If cos(*θ*) = 1, it indicates that the directions of overall change in the two vectors is exactly the same, and the similarity is the strongest. If cos(*θ*) = 0, it indicates that the difference between the two vectors is large, and the similarity is weak. In the case, the cosine similarities among *CI*, *EAB*, and *TS* are presented in Table 2.

From the comparison of waveform similarities, it can be seen that *EAB* and *TS* have a high degree of waveform similarity, with vector directions being relatively consistent, which aligns with the fact that they are both characteristics of mechanical testing. In contrast, the waveform similarities between *CI*, *TS,* and *EAB* are generally lower, with waveform changes not being entirely consistent, which is related to the fact that *CI* is essentially derived from spectral features. Therefore, the mathematical characteristics and associations of different indicators are related to their specific physical backgrounds. For convenience, this paper primarily characterizes their correlations from a mathematical perspective, while deeper intrinsic associations will be explored in future research.

Due to the differences between accelerated thermal aging tests and natural aging processes, as well as the limited number of test samples, Equations (8)–(10) are only applicable to the results obtained from the set of tests in the paper. More general correlation results require additional experiments and more data support. From a practical perspective, the correlation between near-infrared spectroscopy and macroscopic mechanical characteristics primarily stems from the effects of aging. Identifying appropriate quantitative methods for aging levels will help clarify the general quantitative relationship between near-infrared spectroscopy and macroscopic mechanical characteristics. In addition, different testing methods essentially assess different properties of the material. Although aging can affect these properties, there is no direct correlation between spectral characteristics and mechanical properties. Therefore, the primary research direction in the near future should focus on quantifying the effects of aging on material properties.

Currently, the application of NIR as a replacement for traditional cable-aging tests still faces certain limitations, particularly concerning the reliability, applicability, and boundary conditions of the obtained results. However, these challenges can be progressively mitigated by accumulating a substantial volume of experimental data to establish a comprehensive reference database. Furthermore, with the anticipated integration of artificial intelligence technologies, the identification of key model parameters could be significantly enhanced, enabling the development of more precise and effective diagnostic criteria. This advancement would ultimately promote the broader adoption of NIR in cable-aging assessments.

## 5. Conclusions

This study employed various testing techniques, including FTIR, EAB, and TS, to evaluate the performance of XLPE insulation material used in power cables at temperatures of 105 °C, 135 °C, 155 °C, and 180 °C. In addition, NIR tests were applied to the samples, followed by a comprehensive correlation analysis between the NIR results and those obtained from the FTIR, EAB, and TS tests. The analysis yielded numerical relationships linking the characteristic peaks in the NIR with the values from FTIR, EAB, and TS tests. The specific conclusions are summarized as follows:

(1) The correlation between the NIR and FTIR, EAB, and TS can be quantified with expressions, which indicates that it is possible to obtain the same or similar aging assessment results by replacing FTIR, EAB, and TS tests with NIR.
(2) The test results of FTIR, EAB, and TS all reflect the changes of aging to some degree and exhibit certain similarities. The combined use of these test results can effectively assess the extent of aging.
(3) The absorption peaks of ‘C-H (-CH2-)’ (1730 nm/1764 nm) in the near-infrared spectroscopy can serve as the characteristic peaks of aging, with its amplitude decreasing as the aging time increases, and the curve changes relatively gradually.
(4) The waveform similarity of tests of the same type (EAB and TS) is quite high, and the alternative testing using NIR is likely to only reflect relatively rough macroscopic mechanical characteristics. Accurate test results still require specialized mechanical testing.
(5) There is a certain feasibility in replacing traditional cable-aging tests with NIR. In the future, the introduction of artificial intelligence technology to achieve automatic acquisition and identification of key parameters and indicators may promote its practical application.

## Figures and Tables

**Table 1:** Thermal aging test temperatures and times for XLPE.

Aging Temperatures (°C)	105	135	155	180
Aging stage 1	720 h	168 h	72 h	2 h
Aging stage 2	1440 h	336 h	144 h	8 h
Aging stage 3	2160 h	504 h	216 h	14 h
Aging stage 4	3600 h	672 h	288 h	20 h
Aging stage 5	5040 h	840 h	336 h	26 h
Aging stage 6	8640 h	1176 h	360 h	30 h
Aging stage 7	9360 h	1344 h	432 h	32 h

**Table 2:** Cosine similarities among *CI*, *EAB*, and *TS*.

Aging Temperatures (°C)	105	135	155	180
*CI* vs. *EAB*	0.3222	0.0910	0.0585	0.1177
*CI* vs. *TS*	0.3340	0.1771	0.0807	0.1739
*EAB* vs. *TS*	0.9909	0.9698	0.9870	0.9854

## Data Availability

The original contributions presented in the study are included in the article, further inquiries can be directed to the corresponding authors.

## Abbreviations

The following abbreviations are used in this manuscript:

ATR	Attenuated Total Reflectance
CI	Carbonyl Index
DSC	Differential Scanning Calorimetry
EAB	Elongation at Break
ESR	electron spin resonance
FTIR	Fourier transform infrared spectroscopy
IM	indenter modulus
NIR	near-infrared spectroscopy
SGCC	State Grid Corporation of China
TS	tensile strength
XLPE	cross-linked polyethylene
XRD	X-ray diffraction
